# Impact of mental resilience and perceived immune functioning on the severity of alcohol hangover

**DOI:** 10.1186/s13104-018-3659-0

**Published:** 2018-07-31

**Authors:** Aurora J. A. E. van de Loo, Marith van Schrojenstein Lantman, Marlou Mackus, Andrew Scholey, Joris C. Verster

**Affiliations:** 1 Division of Pharmacology, Utrecht University, Universiteitsweg 99, 3584 CG Utrecht, The Netherlands; 2 Institute for Risk Assessment Sciences (IRAS), Utrecht University, Utrecht, The Netherlands; 3 Centre for Human Psychopharmacology, Swinburne University, Melbourne, Australia

**Keywords:** Alcohol, Hangover, Severity, Mental resilience, Perceived immune functioning

## Abstract

**Objective:**

Recent research comparing hangover sensitive drinkers with hangover resistant drinkers has revealed that experiencing alcohol hangovers is associated with significantly poorer self-reported immune functioning (p < 0.0001). No significant difference between the groups was found on mental resilience. The objective of the current survey was to examine the association between hangover severity, perceived immune status, and mental resilience. N = 341 Dutch students, all hangover sensitive drinkers, completed an online survey. The Brief Resilience Scale was completed, and perceived immune functioning and overall hangover severity for their latest past month hangover were assessed.

**Results:**

Students consumed a mean (SD) of 12.3 (5.9) alcoholic drinks the evening before their latest hangover. A significant positive association was found between mental resilience and perceived immune functioning (r = 0.372, p = 0.000). No significant associations of hangover severity were found with mental resilience (r = − 0.010, p = 0.858), or perceived immune functioning (r = − 0.025, p = 0.645). Previous research revealed that hangover resistant and hangover sensitive drinkers report having significantly different levels of immune functioning, and that the immune system is involved in the development of alcohol hangover. These findings suggest that levels of mental resilience and perceived immune functioning are not related to the severity of hangovers in hangover sensitive drinkers.

## Introduction

The alcohol hangover is defined as the combination of mental and physical symptoms, experienced the day after a single episode of heavy drinking, starting when blood alcohol concentration approaches zero [1]. Whereas the majority of drinkers experiences hangovers following an evening of heavy alcohol consumption, a minority of drinkers claim to be hangover resistant [2, 3]. Several studies have compared the characteristics of these two groups of drinkers [4, 5], in order to further elucidate the pathology of the alcohol hangover. This is a potentially important avenue of research as individual differences in the typology of alcohol hangover may help to understand the pathology of alcohol hangover [6, 7], and identify factors that may aggravate or lessen hangover severity [8].

A recent study looked into biopsychological characteristics of hangover sensitive and hangover resistant drinkers, including mental resilience and perceived immune status [9, 10].

Mental resilience enables recovery from stress and the capacity to face the next stressor with optimism [11, 12]. Hence, this trait is considered to have a positive effect on mental and physical health [13, 14]. Also outside the context of alcohol hangover, van Schrojenstein Lantman et al. demonstrated the existence of interrelationships between mental resilience, self-reported immune functioning, and health [15]. In a first analysis [9] we compared mental resilience of hangover resistant drinkers and hangover sensitive drinkers. It was hypothesized that alcohol is an example of a stressor, and that hangover resistance of certain drinkers may be explained by having higher levels of mental resilience than hangover sensitive drinkers. This hypothesis was not supported. Specifically the data show that hangover sensitive drinkers did not significantly differ from hangover resistant drinkers on levels of mental resilience.

In a second analysis [10], we examined whether hangover resistant drinkers and hangover sensitive drinkers differ on levels of self-reported immune functioning. It was argued that if hangover sensitive drinkers report significantly poorer immune status this could explain why they experience relatively more/worse hangovers than hangover resistant drinkers. The data indeed revealed that hangover sensitive drinkers had significant poorer self-reported immune functioning scores compared to hangover resistant drinkers (p = 0.0001). Thus, hangover frequency and symptomatology appears to be associated with having a poorer baseline immune status.

It is important to stress that the study only differentiated between hangover resistant drinkers and hangover sensitive drinkers. No information was gathered about a possible relationship between mental resilience and perceived immune status with the severity of alcohol hangovers. However, it could be argued that, among hangover sensitive drinkers, hangovers are more severe in those drinkers who report a poorer perceived immune status, whereas lower levels of mental resilience would not affect hangover severity. This hypothesis was investigated in the current analysis.

## Main text

### Methods

Data from an online survey [1] was used to examine our hypothesis. The online survey was designed using www.surveymonkey.com, advertised via Facebook.com, and conducted in December 2016. Subjects who completed the survey, and gave permission to be approached for follow-up research (N = 950), were invited by email to complete a follow up survey. Data from this follow-up survey was used for the current analysis. Subjects were included if they were Dutch university students, aged 18–30 years old, and reported to be sensitive to having hangovers. The and took about 10 min to complete.

#### Alcohol consumption and hangover severity

In addition to demographic data, weekly alcohol consumption was assessed, as well as the amount of alcohol consumption the day before the past month latest hangover. Information about weight and gender was used to calculate an estimated peak Blood Alcohol Concentration (e-pBAC) for this drinking occasion, using an adapted Widmark equation [16]. Overall hangover severity was rated on an 11-point scale ranging from 0 (absent) to 10 (extreme) [4].

#### Perceived immune functioning

Perceived immune functioning was assessed with a scale ranging from 0 (very poor) to 10 (excellent) [15]. Previous studies revealed significant correlations of 1-item perceived immune functioning scores with mental resilience [15], autism traits [17], and the Immune Function Questionnaire [15].

#### Mental resilience

Mental resilience was determined with the Brief Resilience Scale (BRS) [18]. The BRS consists of 6 items and measures the ability to recover from stress, i.e. to bounce back. BRS items are scored on a 5-point Likert scale ranging from 1 (‘strongly disagree’) to 5 (‘strongly agree’). Higher scores imply higher levels of mental resilience. Previous research showed that BRS scores correlated significantly with various personality characteristics, psychological coping strategies and health [15, 18].

#### Statistical analysis

Data were analyzed using SPSS, version 24. Means and standard deviations (SD) of each variable were computed. Individual scores on mental resilience, perceived immune functioning, hangover severity, e-pBAC, and number of alcoholic drinks consumed were correlated using nonparametric Spearman’s rho correlations. Correlations were considered significant if p < 0.05. Data from men and women was compared using independent t-tests or a nonparametric Independent Samples Man-Whitney *U* test.

## Results

N = 341 subjects completed the survey. Their mean (SD) age was 20.9 (2.4) years old, and N = 156 of them was men (45.7%). A summary of their demographics and other assessments is given in Table 1.

**Table 1:** Summary of assessments

	Total	Men	Women	p value
N	341	156	185	
Demographics				
Age (years)	20.9 (2.4)	21.3 (2.6)	20.7 (2.1)	0.023*
Weight (kg)	72.4 (12.6)	78.2 (12.2)	67.5 (10.7)	0.000*
Usual number of alcoholic drinks per week	12.8 (10.3)	16.3 (10.8)	9.8 (8.9)	0.000*
Perceived immune functioning	7.4 (1.3)	7.7 (1.3)	7.1 (1.3)	0.007*
Mental resilience	21.0 (4.0)	21.6 (4.2)	20.5 (3.8)	0.012*
Latest hangover occasion				
Number of alcoholic drinks consumed	12.6 (5.9)	14.4 (6.4)	11.1 (4.8)	0.000*
Duration of the drinking session (h)	5.7 (2.1)	6.1 (2.1)	5.5 (2.0)	0.006*
e-pBAC (%)	0.19 (0.1)	0.17 (0.1)	0.21 (0.1)	0.022*
Overall hangover severity	6.0 (1.9)	6.0 (1.9)	6.0 (1.9)	0.809

Mean (SD) are shown. Significant gender differences (p < 0.05) are indicated by asterisk

*e-pBAC* estimated peak blood alcohol concentration

Table 1 shows that men and women differ significantly on all demographic assessments. Also alcohol consumption levels, both regular and on the latest past month heavy drinking occasion resulting in a hangover, are significantly higher in men compared to women. Hangover severity scores, for the latest heavy drinking occasion, did not significantly differ between men and women.

A significant association was found between alcohol hangover severity and the number of alcoholic drinks that were consumed on the latest past month drinking session resulting in a hangover (r = 0.158, p = 0.003) and the duration of drinking (r = 0.189, p = 0.000). The association with e-pBAC was not significant (r = 0.097, p = 0.073). Hangover severity did not correlate significantly with usual weekly alcohol consumption (r = 0.046, p = 0.399), and also no significant associations of hangover severity were found with age (r = 0.046, p = 0.392), body weight (r = 0.021, p = 0.693), mental resilience (r = − 0.010, p = 0.858), or perceived immune functioning (r = − 0.025, p = 0.645).

The number of alcoholic drinks consumed on the latest drinking session resulting in a hangover correlated significantly positive with mental resilience (r = 0.171, p = 0.002) and perceived immune functioning (r = 0.115, p = 0.034). These associations were not significant for the duration of the drinking session. Usual weekly alcohol intake correlated significantly with mental resilience (r = 0.117, p = 0.032), but not with perceived immune functioning (r = 0.070, p = 0.903). eBAC did not correlated significantly with mental resilience (r = 0.094, p = 0.084) and perceived immune functioning (r = − 0.009, p = 0.868). Finally, a significant positive association was found between mental resilience and perceived immune functioning (r = 0.372, p = 0.000).

## Discussion

Although hangover resistant and hangover sensitive drinkers differ significantly in self-reported immune functioning (p = 0.000) [10], the current findings show that levels of perceived immune functioning do not affect the severity of hangovers within a hangover sensitive cohort. Thus, whereas hangover sensitive drinkers typically report having lower immune status compared to hangover resistant drinkers, having a better perceived immune functioning does not imply that one has less severe hangovers. Also, at least within this cohort, the level of mental resilience was not significantly associated with hangover severity.

Research into biopsychological factors that may influence the presence and severity of alcohol hangovers is scarce. To date, these studies suggest that immune status can have an influence on the presence of hangovers, but not on their severity. This warrants further investigation. For example, hangover susceptibility can be compared in drinkers who claim to be hangover sensitive and resistant by assessing biomarkers of immune status (e.g. cytokines in blood or saliva). Clearly, other factors impacting alcohol consumption may have an influence on hangover susceptibility and these merit further investigation, as do immune status-related individual differences. Studies that experimentally manipulate immune status, together with biomarker assessments, may help to further increase our knowledge on the pathology of the alcohol hangover.

Notwithstanding the common limitations of survey research per se (e.g., recall bias), the current literature suggest a role of the immune system in the development of the alcohol hangover, and further research is necessary to elucidate this interaction.

## Limitations

The current self-reported data may suffer from common limitations experienced in survey research such as recall bias or socially desirable answering. However, as the survey was anonymous, this reduced the possibility of obtaining socially desirable answering. Also, if recall bias would have played a role there is no reason to assume that this would have a differential impact for hangover resistant and hangover sensitive drinkers. Finally, the study was conducted among young adults, which makes it unclear to what extend the results are generalizable to other age groups.

## References

[1] Van Schrojenstein Lantman M, Mackus M, van de Loo AJAE, Verster JC (2016). Development of a definition for the alcohol hangover: consumer descriptions and expert consensus. Curr Drug Abuse Rev.

[2] Verster JC, de Klerk S, Bervoets AC, Kruisselbrink LD (2013). Can hangover immunity really be claimed?. Curr Drug Abuse Rev..

[3] Kruisselbrink LD, Bervoets AC, de Klerk S, van de Loo AJAE, Verster JC (2017). Hangover resistance in a Canadian university student population. Addict Behav Rep..

[4] Hogewoning A, Van de Loo A, Mackus M, Raasveld SJ, De Zeeuw R, Bosma ER, Bouwmeester NH, Brookhuis KA, Garssen J, Verster JC (2016). Characteristics of social drinkers with and without a hangover after heavy alcohol consumption. Subst Abuse Rehabil..

[5] Mackus M, van Schrojenstein Lantman M, Van de Loo AJAE, Brookhuis KA, Kraneveld AD, Garssen J, Verster JC (2018). Alcohol metabolism in hangover sensitive versus hangover resistant social drinkers. Drug Alcohol Dep..

[6] Penning R, van Nuland M, Fliervoet LAL, Olivier B, Verster JC (2010). The pathology of alcohol hangover. Curr Drug Abuse Rev..

[7] Tipple CT, Benson S, Scholey A (2016). A review of the physiological factors associated with alcohol hangover. Curr Drug Abuse Rev..

[8] Prat G, Adan A, Sánchez-Turet M (2009). Alcohol hangover: a critical review of explanatory factors. Hum Psychopharmacol.

[9] Van Schrojenstein Lantman M, van de Loo AJAE, Mackus M, Kraneveld AD, Brookhuis KA, Garssen J, Verster JC (2018). Susceptibility to alcohol hangovers: not just a matter of being resilient. Alcohol Alcohol.

[10] Van de Loo AJAE, Mackus M, Van Schrojenstein Lantman M, Kraneveld AD, Brookhuis K, Garssen J, Scholey A, Verster JC (2018). Susceptibility to alcohol hangovers: the association with self-reported immune status. Int J Environ Res Publ Health..

[11] Shastri PC (2013). Resilience: building immunity in psychiatry. Ind J Psychiatry.

[12] Hu T, Zhang D, Wang J (2015). A meta-analysis of the trait resilience and mental health. Personality and individual differences. Pers Ind Differ.

[13] Tugade MM, Fredrickson BL, Barrett LF (2004). Psychological resilience and positive emotional granularity: examining the benefits of positive emotions on coping and health. J Pers.

[14] Schure MB, Odden M, Goins RT (2013). The association of resilience with mental and physical health among older American Indians: the native elder care study. Am Indian Alsk Native Ment Health Res.

[15] Van Schrojenstein Lantman M, Otten LS, Mackus M, de Kruijff D, van de Loo AJAE, Kraneveld AD, Garssen J, Verster JC (2017). Mental resilience, perceived immune functioning, and health. J Multidisc Healthc..

[16] Watson PE, Watson ID, Batt RD (1981). Prediction of blood alcohol concentrations in human subjects. Updating the Widmark Equation. J Stud Alcohol.

[17] Mackus M, de Kruijff D, Otten LS, Kraneveld AD, Garssen J, Verster JC (2017). Differential gender effects in the relationship between perceived immune functioning and autism spectrum disorder scores. Int J Environ Res Publ Health..

[18] Smith BW, Wiggins K, Dalen J, Bernard J (2008). Brief resilience scale: assessing the ability to bounce back. Int J Behav Med..

